# Protection of Omicron Bivalent Vaccine, Previous Infection, and Their Induced Neutralizing Antibodies Against Symptomatic Infection With Omicron XBB.1.16 and EG.5.1

**DOI:** 10.1093/ofid/ofae519

**Published:** 2024-09-06

**Authors:** Shohei Yamamoto, Kouki Matsuda, Kenji Maeda, Tetsuya Mizoue, Kumi Horii, Kaori Okudera, Tomofumi Tan, Yusuke Oshiro, Natsumi Inamura, Takashi Nemoto, Junko S Takeuchi, Maki Konishi, Haruhito Sugiyama, Nobuyoshi Aoyanagi, Wataru Sugiura, Norio Ohmagari

**Affiliations:** Department of Epidemiology and Prevention, Center for Clinical Sciences, National Center for Global Health and Medicine, Tokyo, Japan; Division of Antiviral Therapy, Joint Research Center for Human Retrovirus Infection, Kagoshima University, Kagoshima, Japan; Division of Antiviral Therapy, Joint Research Center for Human Retrovirus Infection, Kagoshima University, Kagoshima, Japan; Department of Refractory Viral Infection, Research Institute, National Center for Global Health and Medicine, Tokyo, Japan; Department of Epidemiology and Prevention, Center for Clinical Sciences, National Center for Global Health and Medicine, Tokyo, Japan; Infection Control Office, Center Hospital of the National Center for the Global Health and Medicine, Tokyo, Japan; Infection Control Office, Kohnodai Hospital of the National Center for the Global Health and Medicine, Chiba, Japan; Department of Laboratory Testing, Center Hospital of the National Center for the Global Health and Medicine, Tokyo, Japan; Department of Laboratory Testing, Center Hospital of the National Center for the Global Health and Medicine, Tokyo, Japan; Department of Laboratory Testing, Center Hospital of the National Center for the Global Health and Medicine, Tokyo, Japan; Department of Laboratory Testing, Center Hospital of the National Center for the Global Health and Medicine, Tokyo, Japan; Department of Academic-Industrial Partnerships Promotion, Center for Clinical Sciences, National Center for Global Health and Medicine, Tokyo, Japan; Department of Epidemiology and Prevention, Center for Clinical Sciences, National Center for Global Health and Medicine, Tokyo, Japan; Center Hospital of the National Center for the Global Health and Medicine, Tokyo, Japan; Kohnodai Hospital of the National Center for the Global Health and Medicine, Chiba, Japan; Center for Clinical Sciences, National Center for Global Health and Medicine, Tokyo, Japan; Disease Control and Prevention Center, National Center for Global Health and Medicine, Tokyo, Japan

**Keywords:** bivalent vaccine, COVID-19, neutralizing antibody, previous infection, protection

## Abstract

**Background:**

Data are limited on the protective role of the Omicron BA bivalent vaccine, previous infection, and their induced neutralizing antibodies against Omicron XBB.1.16 and EG.5.1 infection.

**Methods:**

We conducted a nested case-control analysis among tertiary hospital staff in Tokyo who had received ≥3 doses of COVID-19 vaccines and donated blood samples in June 2023 (1 month before the Omicron XBB.1.16 and EG.5.1 wave). We identified 206 symptomatic cases between June and September 2023 and selected their controls with 1:1 propensity score matching. We examined the association of vaccination, previous infection, and preinfection live virus neutralizing antibody titers against Omicron XBB.1.16 and EG.5.1 with the risk of COVID-19 infection.

**Results:**

Previous infection during the Omicron BA- or XBB-dominant phase was associated with a significantly lower infection risk during the XBB.1.16 and EG.5.1–dominant phase than infection-naive status, with 70% and 100% protection, respectively, whereas Omicron BA bivalent vaccination showed no association. Preinfection neutralizing titers against XBB.1.16 and EG.5.1 were 39% (95% CI, 8%–60%) and 28% (95% CI, 8%–44%) lower in cases than matched controls. Neutralizing activity against XBB.1.16 and EG.5.1 was somewhat detectable in the sera of individuals with previous infection but barely detectable in those who were infection naive and received the Omicron bivalent vaccine.

**Conclusions:**

In the era when the Omicron XBB vaccine was unavailable, the Omicron BA bivalent vaccine did not confer the neutralizing activity and protection against Omicron XBB.1.16 and EG.5.1 symptomatic infection. The previous infection afforded neutralizing titers and protection against symptomatic infection with these variants.

## Teaser

In the era when the Omicron XBB vaccine was unavailable, the Omicron bivalent vaccine did not confer neutralizing activity and protection against Omicron XBB.1.16/EG.5.1 symptomatic infection. The previous infection afforded neutralizing titers and protection against symptomatic infection with these variants.

As of 2024, four years after the initial outbreak, the COVID-19 pandemic is still ongoing due to the persistent mutation cycle of SARS-CoV-2. In late 2020, clinical trials showed that COVID-19 vaccination was highly effective in lowering the risk of SARS-CoV-2 infection and severe outcomes [1, 2]. In late 2021, COVID-19 cases rapidly increased due to the Omicron BA subvariants among the vaccinated population. In 2022, an updated Omicron BA bivalent vaccine lowered the risk of Omicron BA infections [3]. In early to mid-2023, the Omicron XBB subvariants (XBB.1.5, XBB.1.16, and EG.5), with multiple spike protein mutations as compared with earlier BA subvariants [4–6], dominated worldwide. Until the Omicron XBB vaccine became available in September 2023, people had to rely on immunity acquired by existing vaccines or prior infections against Omicron XBB subvariants.

In immunologic studies, Omicron bivalent BA vaccines and Omicron BA infection elicited neutralizing activity against XBB.1.5, XBB.1.16, and EG.5 subvariants, albeit to a limited extent [7–9]. In epidemiologic studies, while the Omicron BA bivalent vaccine or previous Omicron BA infection was reported to confer moderate protection against the Omicron XBB.1.5 infection [3, 10, 11], the evidence regarding the protection against Omicron XBB.1.16 and EG.5 infection is limited. In a cohort study of 51 017 US health care workers, previous Omicron infection, not the Omicron bivalent vaccine, was associated with a lower risk of subsequent infection when Omicron XBB.1.16 and EG.5 subvariants were dominant [12]. Quantitative association between vaccine- or infection-acquired neutralizing activity against Omicron XBB.1.16 and EG.5 and the risk of infection with these variants remains elusive.

In June 2023, a month before the Omicron XBB.1.16 and EG.5.1 epidemic in Japan (July–September 2023), we performed a serologic survey among the staff of the National Center for Global Health and Medicine (NCGM), Tokyo, and stored blood samples. This situation prompted us to investigate whether the Omicron bivalent vaccine and previous infection could confer protection against Omicron XBB.1.16 and EG.5.1 infection and whether its induced neutralizing antibody titers could correlate with infection protection.

Here, we examined the protection of the Omicron bivalent vaccine and previous infection against Omicron XBB.1.16 and EG.5.1 infection and compared the live virus and preinfection neutralizing antibody titers between infected cases and controls in a nested case-control study of recipients with ≥3 doses of COVID-19 historical monovalent or Omicron BA bivalent vaccines.

## METHODS

### Study Setting

A repeat serologic study was conducted at the NCGM in Japan in July 2020 to monitor the spread of SARS-CoV-2 infection among staff during the COVID-19 epidemic. The details of this study have been reported elsewhere [13–15]. In summary, we have completed 8 serosurveys as of June 2023, where we measured anti-SARS-CoV-2 nucleocapsid (N) protein antibodies (all serosurveys) and spike protein antibodies (from the second serosurvey onward) for all participants using Abbott and Roche assays, stored serum samples at −80 °C, and collected information on COVID-19–related factors via a questionnaire (vaccination, occupational infection risk, infection prevention practices, behavioral factors, etc). The self-reported vaccination status was validated by objective information from the NCGM Labor Office. Written informed consent was obtained from all participants. This study was approved by the NCGM Ethics Committee (NCGM-G-003598).

### Case-Control Selection

We conducted a nested case-control study among the staff who participated in the eighth survey in June 2023 and had received ≥3 doses of the mRNA COVID-19 vaccine manufactured by Pfizer or Moderna (any of the patterns of historical monovalent vaccine, Omicron BA.1 and wild type bivalent vaccine, and Omicron BA.4/5 and wild type bivalent vaccine; Supplementary Figure 1). Of the 2569 participants, 2409 received ≥3 doses of the mRNA COVID-19 vaccines and donated blood samples. Of those, we excluded 16 participants who lacked information on covariates: body mass index (n = 10), alcohol drinking status (n = 2), living arrangement status (n = 5), adherence to infection prevention practice (n = 3), and infection risk behaviors (n = 2). We further excluded 19 participants with insufficient volume of serum sample (<100 μL), leaving 2374 participants as the base population.

We followed the participants for COVID-19 incidence using the COVID-19 patient records documented by the NCGM Hospital Infection Prevention and Control Unit. Per the NCGM rule, staff should undergo polymerase chain reaction or antigen test for COVID-19 when they have COVID-19–compatible symptoms; if testing positive, they must report the results to the NCGM Hospital Infection Prevention and Control Unit. During the follow-up (June–September 2023), we identified 217 patients with COVID-19. We defined cases as symptomatic SARS-CoV-2 infection. Participants infected after additional vaccination during follow-up were considered cases if the infection occurred within 14 days after the vaccination, assuming they were not sufficiently immunized with the additional booster until then. After 11 asymptomatic patients were excluded, 206 were included as cases (Supplementary Figure 1). We selected a control for each case using propensity score matching. The details of the case-control matching algorithm are described in Supplementary Text 1. We randomly selected 50 matched pairs and measured live virus neutralizing antibody titers to compare neutralizing antibodies between the groups.

### Antibody Testing

We measured neutralizing activity against wild type, Omicron XBB.1.16, and Omicron EG.5.1 in the sera of patients and controls by quantifying the serum-mediated suppression of the cytopathic effect of each SARS-CoV-2 strain in HeLa_hACE2-TMPRSS2_ cells [16, 17]. The details of the measurement methods are described in Supplementary Text 2.

We assessed anti-SARS-CoV-2 antibodies in all participants at baseline and retrieved data for the case-control pairs. We quantitatively measured the levels of antibodies against the receptor-binding domain (RBD) of the SARS-CoV-2 spike protein using the AdviseDx SARS-CoV-2 IgG II assay (Abbott; ie, anti-RBD immunoglobulin G [IgG]) and Elecsys Anti-SARS-CoV-2 S (Roche; ie, anti-RBD total). We chose the 2 anti-RBD assays with Abbott and Roche because of their established reputation and frequent application in the research field, making it easier to compare with other studies. We also qualitatively measured antibodies against the SARS-CoV-2 N protein using the SARS-CoV-2 IgG assay (Abbott) and Elecsys Anti-SARS-CoV-2 (Roche).

### Previous Infection Status at Baseline

Previous infection was defined as a self-reported history of COVID-19 at baseline (confirmed against in-house COVID-19 registry) or anti-N seropositivity with either assay (Roche ≥1.0 cutoff index or Abbott ≥1.40 signal to cutoff) at any of the first through eighth surveys (July 2020–June 2023). We defined participants with no history of COVID-19 but seropositivity on N antibodies as undiagnosed infection [18]. We defined phases of previous infection referring to the timing of the last diagnosis: pre-Omicron (predominated by wild type, Alpha, and Delta strains; February 2020–December 2021), Omicron BA (dominated by BA.1, BA.2, and BA.5 subvariants; January 2022–March 2024), and Omicron XBB (primarily XBB.1.5 and XBB.1.16 subvariants; April 2024–June 2024).

### Statistical Analysis

We used conditional logistic regression while accounting for the matched design to examine the association of vaccination status (doses of any COVID-19 vaccines and dose of Omicron BA vaccines) and previous infection status with SARS-CoV-2 infection risk. We used a generalized estimating equation (GEE) with group assignment (case or control) and a robust variance estimator to compare the interval from the last vaccination or COVID-19 diagnosis to baseline blood sampling. To examine the difference in preinfection antibody levels between cases and controls, we compared the log-transformed titers of neutralizing antibodies (wild type, Omicron XBB.1.16, and Omicron EG.5.1) and anti-RBD antibodies (IgG and total) between matched pairs using a GEE model with group assignment and a robust variance estimator. Then, we back-transformed and presented these values as geometric mean titers (GMTs) with 95% CIs. For a sensitivity analysis, we repeated the GEE analysis by restricting matched pairs to infection-naive pairs at baseline (ie, case and matched controls had no history of COVID-19 and were negative on anti-N assays). We used the Kruskal-Wallis test to compare the neutralizing titers across vaccination status (historical monovalent vaccine only or the historical monovalent plus Omicron bivalent vaccines) and previous infection status (infection naive or previously infected). To examine the difference in neutralizing titers across the timing of previous infection, we used a linear regression model while adjusting age, sex, a history of Omicron bivalent vaccination, and the interval between the last vaccination and blood sampling. For the analyses of neutralizing antibody titers, values below the limit of detection (50% neutralization titer [NT_50_] <40) were given the limit of detection value. Statistical analyses were performed with Stata version 18.0 (StataCorp), and graphics were generated with Prism version 9 (GraphPad). All *P* values were 2-sided, and statistical significance was set at *P* < .05.

## RESULTS

### Distribution of Circulating SARS-CoV-2 Variants During Follow-up

**Figure 1.**
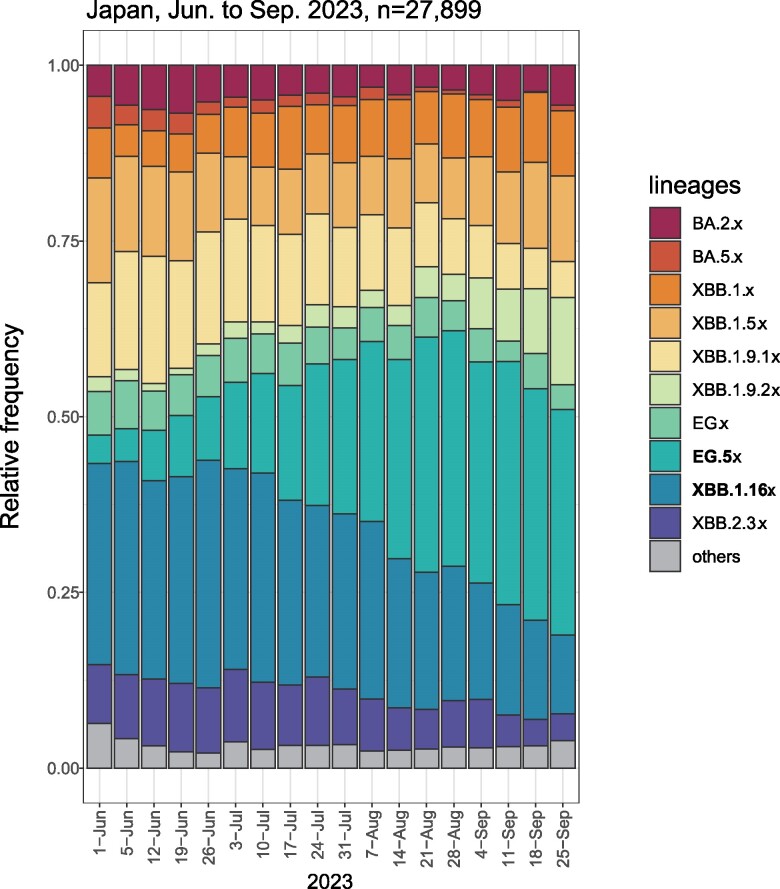
Relative frequency of circulating SARS-CoV-2 variants from June to September 2023 in Japan. The distribution of SARS-CoV-2 lineages in Japan during the study period (June–September 2023) was analyzed by using all domestic genome sequences registered in the GISAID EpiCov database (https://gisaid.org). For lineage analysis, the extracted sequences (n = 27 899) were applied to Phylogenetic Assignment of Named Global Outbreak Lineages version 4.3.1 with pangolin-data 1.25.1.

Figure 1 shows the distribution of SARS-CoV-2 lineages in Japan during the study period (June–September 2023), as analyzed by all domestic genome sequences registered in the GISAID EpiCov database (https://gisaid.org). During the study period, 27 899 samples were extracted for sequences, and the most frequent subvariants were Omicron XBB variants, with a relative frequency of 91%. According to subvariants, the most frequent were Omicron XBB.1.16 (23%) and Omicron EG.5 (22%). From June to September 2023, the relative frequency of Omicron XBB.1.16 decreased (28% to 18%), while that of Omicron EG.5 increased (13% to 31%).

### Baseline Characteristics Before and After Propensity Score Matching

We ascertained 206 symptomatic breakthrough infection cases during the follow-up in the before-matching cohort, with an incidence rate of 12.7 per 10 000 person-days. Cases were younger and more likely to be female and nurses than the controls in the before-matching cohort (Table 1). After propensity matching with a 1:1 ratio, the 206 matched pairs were well balanced regarding all baseline characteristics.

**Table 1.** Baseline Characteristics Before and After Propensity Score Matching

	Before Matching (n = 2363)			After Matching (n = 412)		
Characteristic	Cases (n = 206)	Controls (n = 2157)	Standardized Difference	Cases (n = 206)	Controls (n = 206)	Standardized Difference
Age, y	36.5 ± 11.8	39.3 ± 12.8	0.23	36.5 ± 11.8	36.8 ± 12.4	0.03
Female	77.7	70.4	0.17	77.7	80.1	0.06
Job						
Doctor	11.7	17.2	0.16	11.7	9.7	0.06
Nurse	44.2	36.4	0.16	44.2	46.1	0.04
Allied health care worker	21.4	14.7	0.17	21.4	21.4	0.00
Researcher	6.3	11.9	0.20	6.3	8.3	0.07
Administrative Staff	13.1	14.2	0.03	13.1	12.6	0.01
Others	3.4	5.6	0.11	3.4	1.9	0.09
Occupational SARS-CoV-2 exposure risk^a^						
Low	59.7	60.4	0.01	59.7	62.1	0.05
Moderate	20.4	21.1	0.02	20.4	21.8	0.04
High	19.9	18.5	0.04	19.9	16	0.10
Body mass index, kg/m^2^	21.6 ± 3.3	21.8 ± 3.3	0.07	21.6 ± 3.3	21.6 ± 3.4	0.01
Comorbid diseases^b^	8.7	8.2	0.02	8.7	7.3	0.05
Immunosuppression^c^	1.9	1	0.08	1.9	2.4	0.03
Tobacco products users^d^	6.8	7.3	0.02	6.8	6.3	0.02
Frequency of alcohol drinking						
None	30.6	32.5	0.04	30.6	30.1	0.01
Occasional	32	27.1	0.11	32	33	0.02
Weekly/daily	37.4	40.5	0.06	37.4	36.9	0.01
No. of households	2 ± 1	2 ± 1	0.04	2 ± 1	2 ± 1	0.01
No. of school-age children^e^						
0	72.8	70.9	0.04	72.8	74.8	0.04
1	13.1	12.7	0.01	13.1	11.7	0.04
≥2	14.1	16.4	0.06	14.1	13.6	0.01
Infection prevention practice score^f^	7 ± 2	7 ± 2	0.04	7 ± 2	7 ± 2	0.03
Spending ≥30 min in the 3Cs without mask						
None	59.2	61.8	0.05	59.2	57.8	0.03
1–5 times	32.5	29.1	0.07	32.5	34.5	0.04
≥6 times	8.3	9	0.03	8.3	7.8	0.02
Having dinner in a group ≥5 people for >1 h						
None	60.7	57.7	0.06	60.7	63.1	0.05
1–5 times	36.4	37.8	0.03	36.4	35.4	0.02
≥6 times	2.9	4.5	0.08	2.9	1.5	0.10

Data are presented as mean ± SD for continuous variables and percentage for categorical variables. An absolute standardized difference <0.10 indicates a relatively small imbalance.

Abbreviation: 3Cs, crowded places, close-contact settings, and confined and enclosed spaces.

^a^Occupational SARS-CoV-2 exposure risk was categorized as low (those not engaged in COVID-19–related work), moderate (those engaged in COVID-19–related work without heavy exposure to SARS-CoV-2), or high (those heavily exposed to SARS-CoV-2).

^b^Comorbid diseases were defined as cancer, cardiovascular disease, diabetes, hypertension, chronic kidney disease, or lung disease.

^c^Immunosuppression was defined as having an immunosuppressive disease or using steroids (except topical or inhaled), immunosuppressants, or anticancer drugs.

^d^Tobacco products include conventional cigarettes and heated tobacco products.

^e^School-age children include those in nurseries, kindergartens, elementary to high school, and university and those with disabilities.

^f^Infection prevention practice score was calculated per the total score of adherences to avoiding the 3Cs, hand washing, wearing a mask, and social distancing, as well as not touching the face, nose, or mouth, by assigning 2 points to *always*, 1 to *often*, and 0 to others (*seldom* and *not at all*).

### Vaccination and Previous Infection Statuses vs Risk of COVID-19

The number of existing mRNA vaccinations and that of the Omicron BA bivalent vaccination were not associated with the risk of COVID-19 (Table 2). In the analysis of any mRNA vaccines, the odds ratios (ORs; 95% CI) of 3 to 6 doses against infection were 1 (reference), 1.01 (.62–1.63), 0.78 (.47–1.30), and 1.04 (.32–3.39), respectively. For the analysis of Omicron BA bivalent vaccines, the ORs (95% CI) of no vaccination (monovalent vaccine only), 1 dose, and 2 doses were 1 (reference), 0.79 (.52–1.20), and 1.40 (.32–6.06). The mean interval days between the last vaccination and baseline blood sampling were not statistically different between cases and controls (287 vs 274 days).

**Table 2.** Association of Vaccination Status, Previous Infection Status, and Preinfection Antibody Titers With the Risk of Symptomatic SARS-CoV-2 Infection

Variables	Cases (n = 206)	Controls (n = 206)	Effect Size (95% CI)
Vaccination status at baseline			
No. of mRNA vaccine doses, %			
3	25.7	23.8	1 [Reference]
4	44.7	41.3	1.01 (.62–1.63)^a^
5	26.2	32.0	0.78 (.47–1.30)^a^
6	3.4	2.9	1.04 (.32–3.39)^a^
No. of Omicron bivalent vaccine doses, %			
0 (monovalent vaccine only)	55.3	50.5	1 [Reference]
1	42.2	48.1	0.79 (.52–1.20)^a^
2	2.4	1.5	1.40 (.32–6.06)^a^
Interval from last vaccination to blood sampling, d, mean (95% CI)	287 (268–305)	274 (255–293)	12.7 (−12.9 to 38.2)^b^
Previous infection status at baseline			
Previous SARS-CoV-2 infection status, %			
Infection naive	74.3	39.8	1 [Reference]
Undiagnosed infection	7.8	16.0	0.27 (.13–.55)^a^
Last diagnosed infection before Omicron waves	1.0	1.9	0.20 (.03–1.21)^a^
Last diagnosed infection during Omicron BA waves	17.0	35.9	0.30 (.18–.50)^a^
Last diagnosed infection during Omicron XBB waves	0	6.3	0.00 (NA)^a^
Interval from last diagnosed infection to blood sampling, d, mean (95% CI)^c^	367 (317–417)	286 (254–318)	81.0 (25.3–136.6)^b^
Antibody titer at baseline, GMT (95% CI)			
Anti-RBD IgG antibody: Abbott, AU/mL	6189 (5365–7141)	11 959 (10 238–13 970)	0.52 (.42–.64)^d^
Anti-RBD total antibody: Roche, U/mL	6858 (5987–7856)	12 559 (10 672–14 779)	0.55 (.44–.68)^d^
Neutralizing antibody, NT_50_^e^			
Wild type	287 (194–423)	497 (332–744)	0.58 (.37–.91)^d^
Omicron BBX.1.16	53 (44–64)	87 (61–124)	0.61 (.40–.92)^d^
Omicron EG.5	41 (40–43)	57 (45–73)	0.72 (.56–.92)^d^
Antibody titer at baseline restricted to infection-naive pairs, GMT (95% CI)			
Anti-RBD IgG antibody: Abbott, AU/mL^f^	4960 (3640–6280)	5433 (3760–7107)	0.91 (.59–1.40)^d^
Anti-RBD total antibody: Roche, U/mL^f^	5611 (4076–7145)	5775 (3458–8091)	0.97 (.57–1.65)^d^
Neutralizing antibody			
Wild type, NT_50_^f^	129 (67–191)	177 (49–305)	0.73 (.32–1.67)^d^
Omicron BBX.1.16, No. (%), >40 NT_50_^g^	0/12 (0)	2/12 (16.7)	…
Omicron EG.5, No. (%), >40 NT_50_^g^	0/12 (0)	0/12 (0)	…

Abbreviations: AU, arbitrary units; GMT, geometric mean titer; NA, not applicable; NT_50_, 50% neutralization titer.

^a^Odds ratio of SARS-CoV-2 infection across exposure groups, estimated by the conditional logistic regression model.

^b^Mean difference between cases and controls, estimated by the generalized estimating equation model.

^c^Analyzed among those with a history of COVID-19 diagnosis (case/control: n = 37/91).

^d^GMT ratio for cases to controls, estimated by the generalized estimating equation model.

^e^Analyzed among 50 matched pairs randomly selected from 206 matched pairs.

^f^Analyzed among 57 infection-naive pairs.

^g^Analyzed among 12 infection-naive pairs.

Previous infection at the Omicron BA or XBB phase, but not the pre-Omicron phase, was significantly associated with a lower risk of SARS-CoV-2 infection during the follow-up (Table 2). When compared with infection-naive status, the ORs (95% CI) of previous infection at the pre-Omicron and Omicron BA waves against infection were 0.20 (.03–1.21) and 0.30 (.18–.50), respectively. No SARS-CoV-2 infection occurred in the group of previous infection at the Omicron XBB wave (OR, 0.00). Undiagnosed infection was also associated with a lower risk of infection than infection-naive status (OR, 0.27; 95% CI, .13–.55). Among the participants with a history of COVID-19, the interval between the last diagnosed infection and baseline blood sampling was statistically longer in cases than controls, with a mean difference of 81 days (95% CI, 25–137).

### Preinfection Antibody Titers Between Cases and Matched Controls

The GMTs of preinfection neutralizing antibodies against wild type, Omicron XBB.1.16, and EG.5.1 were 377, 68, and 49, and their detection rates (>40 NT_50_) were 89%, 28%, and 14%, respectively, among total samples of cases and controls (Supplementary Figure 2).

**Figure 2.**
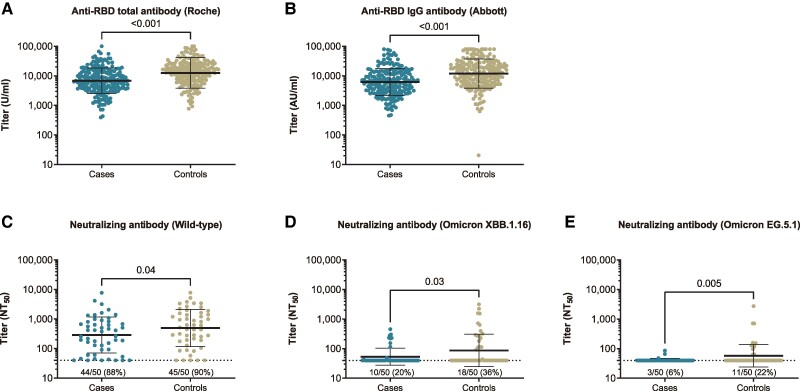
Comparison of the preinfection live virus neutralizing and anti-RBD antibody titers between propensity score–matched cases and controls. *A* and *B*, Among 206 cases with breakthrough infection and 206 matched controls, anti-RBD total antibody titers were measured with Roche reagent, and anti-RBD IgG antibody titers were measured with the Abbott reagent. *C–E*, In addition, the live virus neutralizing antibody titers against wild type, Omicron XBB.1.16, and Omicron EG.5.1 among the 50 matched pairs were randomly selected from 206 matched pairs. In each panel, the horizontal bars indicate the geometric mean titers, and the I-shaped bars indicate the geometric SD. The limit of detection of the neutralizing assay is 40, as shown by dashed horizontal lines. The frequency (percentage) of the upper limit of detection is denoted above the x-axis. *P* values were calculated via the generalized estimating equation model. AU, arbitrary units; IgG, immunoglobulin G; NT_50_, 50% neutralizing titer; RBD, receptor-binding domain.

Preinfection anti-RBD and neutralizing antibody titers were lower in cases than controls. The GEE-predicted GMTs (95% CI) of the anti-RBD IgG antibody on Abbott assay was 6189 AU/mL (5365–7141) for cases and 11 959 AU/mL (10 238–13 970) for controls with a predicted case:control ratio of the titers of 0.52 (95% CI, .42–.64; Table 2, Figure 2). The GMTs (95% CI) of the anti-RBD total antibody on Roche assay were 6858 U/mL (5987–7856) for cases and 12 559 U/mL (10 672–14 779) with a ratio of 0.55 (95% CI, .44–.68). The predicted neutralizing antibody GMTs (95% CI) against wild type (NT_50_) were 287 (194–423) for cases and 497 (332–744) for controls, with a ratio of 0.58 (95% CI, .37–.91). The detection rate of neutralization (≥40 NT_50_) against Omicron XBB.1.16 was lower in cases than controls (20% vs 36%), and the GMTs (95% CI) of Omicron XBB.1.16 were 53 (44–64) for cases and 87 (61–124) for controls. The rate of neutralization detection against Omicron EG.5.1 was lower in cases than controls (6% vs 22%), and the GMTs (95% CI) of Omicron EG.5.1 were 41 (40–43) for cases and 57 (45–73) for controls.

The sensitivity analyses restricted to infection-naive matched pairs indicated that the difference in preinfection anti-RBD and neutralizing antibody titers between cases and controls was attenuated and no longer statistically significant. No infection-naive cases detected neutralization against Omicron XBB.1.16 and EG.5.1.

### Neutralizing Antibody Titers Across Statuses of Omicron Bivalent Vaccination and Previous Infection

**Figure 3.**
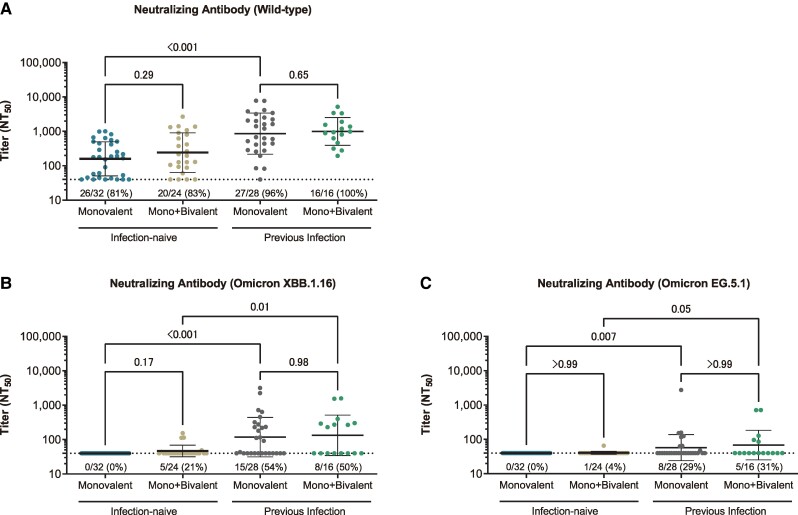
Comparison of the preinfection live virus neutralizing antibody titers across the histories of Omicron bivalent vaccine and previous SARS-CoV-2 infection. *A–C*, Live virus neutralizing antibody titers against wild type, Omicron XBB.1.16, and Omicron EG.5.1 among the 100 samples from 50 matched pairs. In each panel, the horizontal bars indicate the geometric mean titers, and the I-shaped bars indicate the geometric SD. The limit of detection of the neutralizing assay is 40, as shown by dashed horizontal lines. The frequency (percentage) of the upper limit of detection is denoted above the x-axis. *P* values were calculated via the Kruskal-Wallis test. NT_50_, 50% neutralizing titer; RBD, receptor-binding domain.

There were no substantial differences in preinfection neutralizing antibody titers against wild type, Omicron XBB.1.16, and Omicron EG.5.1 among individuals irrespective of their history of Omicron bivalent vaccination (Figure 3). Neutralization against Omicron XBB.1.16 and EG.5.1 was not detected (<40 NT_50_) in all sera from individuals who were infection naive and had never received the Omicron bivalent vaccine. Among those who were infection naive with a history of Omicron bivalent vaccine, only 21% and 4% had detectable neutralizing titers against XBB.1.16 and EG.5.1, respectively.

**Figure 4.**
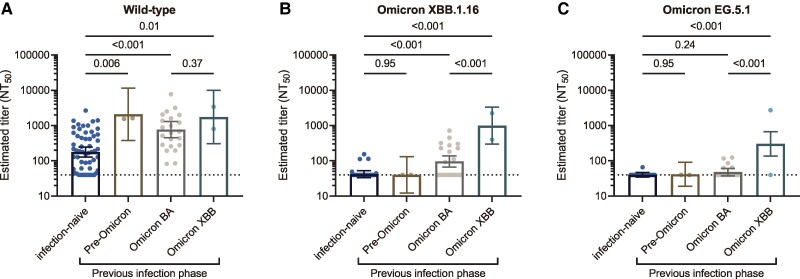
Neutralizing antibody titers across the timing of previous SARS-CoV-2 infection. *A–C*, The bars indicate geometric mean titers of preinfection neutralizing antibodies against wild type, Omicron XBB.1.16, and Omicron EG.5.1 across the timing of the previous infection. Estimates are based on a linear regression model, adjusting age, sex, a history of Omicron bivalent vaccination, and the interval between last vaccination and blood sampling. Error bars indicate 95% CI. The limit of detection of the neutralizing assay is 40, as shown by dashed horizontal lines. *P* values were calculated via the linear regression model. The sample sizes of the infection-naive, pre-Omicron, Omicron BA, and Omicron XBB groups are 58, 2, 22, and 2, respectively. Individuals with undiagnosed infection and their neutralizing titers (n = 18) were not included in this analysis since their infection timing was unclear. We defined each previous infection phase as follows: pre-Omicron, February 2020–December 2021; Omicron BA, January 2022–March 2024; and Omicron XBB, April 2024–June 2024. NT_50_, 50% neutralizing titer.

Participants previously infected had higher preinfection neutralizing antibody titers against wild type, Omicron XBB.1.16, and Omicron EG.5.1 than those who were infection naive (Figure 3). Irrespective of the previous infection phases, wild type neutralizing titers were higher in those with previous infection than those who were infection naive (Figure 4). Those infected during the Omicron XBB periods had the highest neutralizing antibody titers against Omicron XBB.1.16 and EG.5.1 as compared with those infected in the Omicron BA period or earlier. Those infected in the Omicron BA period had statistically higher neutralizing titers against Omicron XBB.1.16 than those who were infection naive but not in titers against Omicron EG.5.1.

## DISCUSSION

From June to September 2023, when Omicron XBB.1.16 and EG.5.1 were predominantly circulating in Japan, previous infection during the Omicron BA and XBB waves was associated with 70% and 100% lower risk of subsequent symptomatic SARS-CoV-2 infection, respectively, while the Omicron BA bivalent vaccination was not associated with the risk of infection in a cohort of health care workers with ≥3 doses of vaccination. The preinfection neutralizing antibody titers against Omicron XBB.1.16 and EG.5.1 were lower in infected cases than in matched controls.

Although the evidence is scarce regarding the protection of previous infections and the bivalent vaccine against Omicron XBB.1.16 and EG.5.1 infection, our findings were similar to a study of US health care workers [12]. In that study, previous Omicron infection was associated with a 60% lower risk of subsequent infection during the dominant waves of Omicron BA.4/5, BQ, or XBB subvariants, whereas the Omicron BA bivalent vaccination was not associated with the risk of infection during the dominant wave of the XBB subvariant. Regarding the protection of previous infection, our study has some strengths over the previous one. We classified previous Omicron infection by the subvariant-specific dominant phase (BA or XBB). In addition, we followed subsequent infections restricted to the dominant phase of XBB.1.16 and EG.5.1. These gaps allowed us to estimate the Omicron BA- and XBB-specific protection against subsequent infections during the XBB.1.16- and EG.5.1-specific waves, and we found that previous infection during the Omicron XBB wave had superior protection against infection during the XBB.1.16- and EG.5.1-dominant phase to those during the Omicron BA wave (100% vs 70% protection).

In spite of much lower preinfection neutralizing titers against XBB.1.16 and EG.5.1 than those against wild type, we found that higher neutralizing antibody titers against Omicron XBB.1.16 and EG.5.1 were associated with a lower risk of SARS-CoV-2 infection when these variants were dominant. Our findings suggest that variant-specific neutralizing antibody titers could correlate with protection against infection with its variant, even within the low titers range. We also found that the prevalence of those with hybrid immunity (vaccination and previous infection) was lower in cases than controls (25% vs 60%) and that individuals with hybrid immunity had higher neutralizing titers against XBB.1.16 and EG.5.1 than those who were infection naive. Similarly, previous studies reported that neutralizing titers against XBB.1.16 and EG.5.1 were higher in vaccinated individuals with a history of infections than those without [8, 9, 19]. These results confirm that the evidence that hybrid immunity confers better protective humoral immunity than vaccination alone, which has been recognized for the risk of infection with Omicron BA or earlier variants [20, 21], can be extended to the risk of XBB.1.16 and EG.5.1 infection.

This study had several strengths. We rigorously matched cases and controls using a propensity score estimated by several factors potentially associated with SARS-CoV-2 infection risk, including occupational SARS-CoV-2 exposure risk, living arrangements, comorbidities, infection prevention practices, and high–infection risk behaviors. Blood samples for antibody testing were obtained before infection (1 month before the Omicron XBB.1.16 and EG.5 epidemic onset). Previous SARS-CoV-2 infection was determined according to the history of COVID-19 diagnosis and results of anti-SARS-CoV-2 N assays, allowing us to identify undiagnosed infections. We measured the neutralizing antibody titers using live viruses. However, limitations should be acknowledged. We defined cases as patients with physician-diagnosed symptomatic COVID-19. Since we did not conduct active surveillance to detect SARS-CoV-2 infection during the follow-up period, the present results may not apply to asymptomatic SARS-CoV-2 infection. Data on virus strain was not available for the present cases; yet, the cases were most likely due to the Omicron XBB variant (including XBB.1.16 and its descendent EG.5), which accounted for >90% of sequenced COVID-19 samples in Japan during the follow-up (June–September 2023; Figure 1).

## CONCLUSION

In the era when Omicron XBB.1.16 and EG.5.1 variants were predominant and the Omicron XBB vaccine was still unavailable in Japan, previous Omicron BA or XBB infection, not Omicron bivalent vaccination, was associated with a lower risk of symptomatic SARS-CoV-2 infection. The preinfection and live virus neutralizing antibody titers against Omicron XBB.1.16 and EG.5.1 were lower in infected cases than in their matched controls. Those with a history of Omicron BA bivalent vaccine had barely detectable neutralizing titers against these variants. Our results highlight the importance of infection prevention practices when the circulating variants had high immune evasion from immunity acquired by existing vaccines.

## Supplementary Material

ofae519_Supplementary_Data

## Supplementary Data

Supplementary materials are available at *Open Forum Infectious Diseases* online. Consisting of data provided by the authors to benefit the reader, the posted materials are not copyedited and are the sole responsibility of the authors, so questions or comments should be addressed to the corresponding author.

## Notes

***Acknowledgments*.** We thank Mika Shichishima for her contribution to data collection and the staff of the laboratory testing department for their contribution to antibody testing.

***Author contributions***. Drs Yamamoto and Mizoue had full access to all data in the study and took responsibility for the integrity of the data and the accuracy of the data analysis. Concept and design: S. Y., K. Matsuda, K. Maeda, T. M., H. S., W. S., N. O. Acquisition, analysis, or interpretation of data: S. Y., K. Matsuda, K. Maeda, K. H., K. O., T. T., Y. O., N. I., T. N., J. S. T., M. K., T. M. Drafting of the manuscript: S. Y., T. M. Critical revision of the manuscript for important intellectual content: S. Y., K. Matsuda, K. Maeda, J. S. T., T. M., H. S., N. A., W. S., N. O. Statistical analysis: S. Y., M. K. Administrative, technical, or material support: S. Y., K. Matsuda, K. Maeda, K. H., K. O., T. T., Y. O., N. I., T. N., J. S. T., M. K., T. M., H. S., N. A., W. S., N. O. Supervision: T. M., N. O.

***Disclaimer*.** The funding entities had no role in the design or conduct of the study; collection, management, analysis, and interpretation of the data; preparation, review, or approval of the manuscript; or the decision to submit the manuscript for publication.

***Patient consent statement*.** Written informed consent was obtained from all the participants. This study was approved by the NCGM Ethics Committee (NCGM-G-003598).

***Financial support*.** This work was supported by the NCGM COVID-19 Gift Fund (grant 19K059 to T. M.); Japan Health Research Promotion Bureau Research Fund (grants 2020-B-09 and 2024-B-01 to T. M.); and National Center for Global Health and Medicine (grants 21A2013D and 23A2020D to T. M. and grant 24A1011 to S. Y.). Abbott Japan and Roche Diagnostics provided reagents for the anti-SARS-CoV-2 antibody assays.
